# Dietary Insulin Index (DII) and Dietary Insulin load (DIL) and Caveolin gene variant interaction on cardiometabolic risk factors among overweight and obese women: a cross-sectional study

**DOI:** 10.1186/s40001-024-01638-5

**Published:** 2024-01-24

**Authors:** Reza Amiri khosroshahi, Atieh Mirzababaei, Leila Setayesh, Reza Bagheri, Mohammad Heidari Seyedmahalleh, Alexei Wong, Katsuhiko Suzuki, Khadijeh Mirzaei

**Affiliations:** 1 Department of Clinical Nutrition, School of Nutritional Sciences and Dietetics, Tehran University of Medical Sciences, Tehran, Iran; 2 Department of Community Nutrition, School of Nutritional Sciences and Dietetics, Tehran University of Medical Sciences (TUMS), Tehran, Iran; 3 Department of Exercise Physiology, University of Isfahan, Isfahan, Iran; 4 Department of Health and Human Performance, Marymount University, Arlington, USA; 5 Faculty of Sport Sciences, Waseda University, 2-579-15 Mikajima, Tokorozawa, 359-1192 Japan

**Keywords:** Dietary insulin index (DII), Dietary insulin load (DIL), Caveolin, Cardiovascular disease

## Abstract

**Background and objective:**

Studies have shown that Caveolin gene polymorphisms (CAV-1) are involved in chronic diseases, such as metabolic syndrome. Moreover, the dietary insulin index (DII) and dietary insulin load (DIL) have been shown to potentially elicit favorable effects on cardiovascular disease (CVD) risk. Therefore, this study sought to investigate the effect of DII DIL and CAV-1 interaction on CVD risk factors.

**Methods:**

This cross-sectional study consisted of 333 overweight and obese women aged 18–48 years. Dietary intakes, DII, and DIL were evaluated using the 147-item food frequency questionnaire (FFQ). Serum profiles were measured by standard protocols. The CAV-1 rs 3,807,992 and anthropometric data were measured by the PCR–RFLP method and bioelectrical impedance analysis (BIA), respectively. Participants were also divided into three groups based on DII, DIL score, and rs3807992 genotype.

**Results:**

This comparative cross-sectional study was conducted on 333 women classified as overweight or obese. Participants with A allele for the caveolin genotype and higher DII score showed significant interactions with high-density lipoprotein (HDL) (*P* for AA = 0.006 and *P* for AG = 0.019) and CRI-I (*P* for AA < 0.001 and *P* for AG = 0.024). In participants with AA genotype and greater DII score, interactions were observed in weight, systolic blood pressure (SBP), diastolic blood pressure (DBP), total cholesterol, CRI-II, fat-free mass (FFM), and skeletal muscle mass (SMM) (*P* < 0.079). Those with higher DIL scores and AA genotype had higher weight (*P* = 0.033), FFM (*P* = 0.022), and SMM (*P* = 0.024). In addition, DIL interactions for waist/hip ratio (WHR), waist circumference (WC), triglyceride (TG), CRI-I, and body fat mass (BFM) among individuals with AA genotype, while an HDL interaction was observed in individuals with AG and AA (*P* < 0.066).

**Conclusion:**

The findings of the present study indicate that people who carry the caveolin rs3807992 (A) allele and have greater DII and DIL scores are at higher risk for several cardiovascular disease and metabolic syndrome biomarkers. These results highlight that diet, gene variants, and their interaction, should be considered in the risk evaluation of developing CVD.

## Introduction

Obesity is a chronic disease whose global prevalence has nearly tripled in the last four decades, affecting more women than men in developed and developing countries [1–3]. The obese population includes metabolically healthy and metabolically unhealthy individuals [4]. Indeed, it is not clear whether obesity causes metabolic syndrome as it is also observed in lean people [5]. However, obesity is related to high levels of oxidative stress, which plays an imperative role in the pathogenesis of numerous diseases [6–8], and obesity and insulin resistance (IR) are the factors of the metabolic syndrome that contribute the most its relationship with oxidative stress [6]. Increased IR and oxidative stress in obesity result from changes in several factors, including dietary patterns and genetic backgrounds [9–12]. Dietary patterns are key components associated with a higher incidence of obesity, particularly those containing fast-absorbing carbohydrates, which increase insulin concentrations attributed to hyperinsulinemia and IR [13]. Two proven methods to characterize higher insulin concentrations in serum are the dietary insulin load (DIL) and dietary insulin index (DII), which are also associated with obesity and metabolic syndrome risk factors, indicating that those with a higher score of the aforementioned indexes have greater insulin concentrations [14–16].

Regardless of carbohydrate intake, other macronutrients (proteins and fats) are also involved in insulin secretion [17]. Although they do not raise glucose concentrations as much, they increase fructose, some amino acids, and fatty acids, enhancing insulin secretion [18, 19]. Recently, the food insulin index (FII) has been introduced to measure the insulin response to foods [20]. Indeed, FII is a more accurate way to predict postprandial insulin secretion than other methods [20]. Using the FII, the insulin response to the diet indicated by DII and DIL can be calculated [21, 22]. This is important as high DII and DIL scores, especially in women, are associated with an increased incidence of obesity [14] and metabolic syndrome [16]. Research to date has also shown important associations between DII and DIL with certain glycemic, lipid, and inflammatory markers. For instance, the DIL score was positively associated with fasting blood glucose (FBG) and C-reactive protein (CRP) in older men [23]. In adolescents, a higher DII score was associated with higher IR and food cravings [24]. Moreover, both the DII and DIL are associated with plasma lipids (positively related to triglycerides and inversely related to HDL), especially in obese individuals [25]. The above-mentioned associations reinforce the importance of these dietary indices in populations with excess body fat and metabolic abnormalities.

Several genomes may have synergic effects with dietary patterns leading to a higher prevalence of obesity. Small pits 60–80 nm in diameter are found in the plasma membrane called caveola [26]. The caveola can be found in particular tissues such as adipocytes, vascular endothelial cells, muscle cells, epithelial cells, and fibroblasts [27]. Caveola has several functions, including regulating cholesterol and lipid metabolism, cellular signaling, mechanical protection, endocytosis, and a significant increase in cell surface area [28, 29]. The role of the caveolin gene on metabolic status can be explained by increasing the expression of CAV-1 mRNA in visceral and subcutaneous adipose tissue that is associated with obesity and its related disorders, such as type two diabetes (T2D) [30]. The association of CAV-1 rs3807992 and metabolic syndrome has been confirmed through the effects of CAV-1 on visceral fat and IR [31, 32]. CAV-1 and CAVIN-1 mRNA are highly expressed in visceral and subcutaneous adipose tissues, particularly among obese individuals, which may be associated with dyslipidemia [30], and atherosclerosis [33]. A high-fat diet increases caveolin expression [27], which can affect the insulin pathway [34, 35]. The relationship between CAV-1 and high-fat diets has been shown in knockout mice. The available investigations showed that mice with polymorphisms in this gene had resistance to weight gain, hyperinsulinemia, and accumulation of epididymal fat following a high-fat diet [36–38]. These findings indicate that caveolin gene polymorphisms are closely related to diets and metabolic factors in an obesity-promoting environment. Therefore, evaluating the associations between diet-dependent insulin response indices, metabolic components, and caveolin gene polymorphisms may be clinically relevant to employing successful prevention and treatment strategies in overweight and obese populations.

To date, no study has investigated the association of caveolin gene polymorphisms with DII and DIL. Given the role of caveolin in insulin signaling pathways, the present study aimed to investigate the interaction between caveolin genotypes with DII and DIL on metabolic components and body composition indices in overweight and obese women. The recent research underscores the importance of understanding the interplay between dietary inflammatory index (DII), caveolin-1 (CAV-1) expression, and metabolic markers. This study extends these insights by examining their relationships in a specific population, offering novel perspectives for dietary interventions. The integration of genetic factors like CAV-1 with dietary patterns provides a more comprehensive understanding of metabolic health in women, potentially guiding personalized dietary recommendations and management strategies for obesity and related metabolic disorders. Evaluation of several dietary macronutrients and micronutrients was also performed as these may be involved in insulin signaling and secretion.

## Methods

### Study population

A cross-sectional of 333 overweight and obese (body mass index [BMI]: 25–40 kg/m^2^) premenopausal (18–48 years) women who were referred to the health centers in Tehran were recruited as participants. Women who had a history of acute or chronic illnesses such as hypertension, cardiovascular diseases, diabetes, kidney disease, liver disease, cancers, and thyroid diseases, were pregnant or breastfeeding, smokers, regular alcohol consumers, and those who followed a diet with less than 800 kcal and more than 4,200 kcal were excluded from the study. Moreover, those who took medications or dietary supplements were also excluded. This study was conducted according to the Declaration of Helsinki. All procedures involving human participants were approved by the ethics committee of the Tehran University of Medical Sciences (IR.TUMS.VCR.REC.1398.142). Written informed consent was obtained from all participants.

The screening of potential participants took place between January 2019 and December 2020. We screened a total of 410 overweight and obese women. Of these, only 333 qualified for inclusion, as 77 individuals were excluded for reasons such as age (*n =* 7), pregnancy or lactation (*n =* 10), recent changes in diet (*n =* 8), and other chronic diseases (*n =* 29). Furthermore, subjects with missing data for biomarkers or covariates (*n =* 10), those who did not answer more than 70 questions (*n =* 3) on the FFQ, and those who reported an overall total daily energy intake outside the range of 800–4200 kcal (*n =* 10) were not included in the statistical analysis.

### Anthropometric assessment and body composition

Anthropometrics and body composition measurements took place between 8–9 am after 12 h of overnight fasting. Participants were asked to avoid any strenuous physical activity for 72 h before the assessment. Furthermore, 30 min before the test, participants were asked to urinate (void) completely and avoid consuming water. Weight was measured using a digital scale (Seca 711; Seca, Hamburg, Germany) to the nearest 0.1 kg. Height was measured using a wall-mounted stadiometer (Seca 711; Seca, Hamburg, Germany). Waist circumference (WC) and hip circumference (HC) were determined at both the smallest and largest girths using standard anthropometric guidelines [39]. The waist-to-hip ratio (WHR) was obtained by dividing the waist circumference by the hip circumference. As a final point, we calculated BMI by dividing weight (kg) by the square of height (meters) [BMI = weight/height (kg/m^2^)]. Obesity and overweight were defined as BMI 30–40 kg/m^2^ and BMI 25–29.9 kg/m^2^, respectively. A multi-frequency bioelectrical impedance analyzer, InBody 770 scanner (test–re-test reliability: 0.980) according to manufacturer guidelines.

### Biochemical assessments

Blood samples (10 mL) were taken following an overnight fast (12 h). The serum was separated by centrifuging and stored at a temperature of − 80 °C until the analysis was carried out. All measurements were taken at the nutrition laboratory of TUMS. Commercial kits (Pars Azmoon, Iran) were used to measure lipid profile and glucose. The enzyme-linked immunosorbent assay (ELISA) method (Human insulin ELISA kit, DRG Pharmaceuticals, GmbH, USA) was used to measure serum insulin concentrations.

### Castelli risk indices I and II calculation

Castelli risk indices I (CRI-I) and Castelli risk indices II (CRI- II) were calculated by following the formula (TC/HDL-C) and (LDL-C / HDL-C), respectively [40].

### Blood pressure measurement

Blood pressure was measured using an automated BP monitor (Omron) after ten minutes of sitting. Two measurements at 1-min intervals were collected and averaged.

### Dietary measurements

To assess participants’ dietary intake and nutritional status over the past year, a semi-quantitative food frequency questionnaire (semi-FFQ) was used. This process was designed based on the Willett study that included 147 food items and standard serving sizes for each nutrient [41]. The reliability and validity of this FFQ from common Iranian foods have been previously described [42]. Household measures were used to convert the size of food consumption into grams [43]. Trained nutritionists filled out questionnaires. To find out the participants' daily intake of each food, the Nutritionist 4 software was used.

### Calculation of DII and DIL

The FII was defined as The area under the curve, representing the food insulin response in a portion of 1000 kJ (239 kcal) of energy over 2 h divided by the area under the reference food curve over 2 h with the same amount of energy [18]. To determine the insulin index of each food in the semi-FFQ, we used the methods outlined in Bell's thesis [44]. For some foods that were exclusively in our questionnaire, we used the insulin index of similar foods described by the Bell study [44]. The insulin load of each food was first calculated separately by the following formula: Insulin index of each food × energy content of food/1 g × amount of food consumption (g/d). Insulin loads of the foods consumed in one day were added together, and the DIL was calculated. DII was obtained by dividing DIL by total energy consumption.

### Genotyping

To determine the different genotypes of CAV-1 polymorphisms, DNA was extracted from serum, and then Polymerase Chain Reaction (PCR-R) Restriction Fragment Length Polymorphism of PCR products (RFLP) technique was used to examine cav 1 polymorphisms. Contrast primers were used in PCR: F:3′AGTATTGACCTGATTTGCCATG5′R:5′GTCTTCTGGAAAAAGCACATGA-3′0.1 μl of extracted DNA, 1 μl of Forwarding primers, 1 μl Revers primers, 7 μl of distilled water and 10 μl of Taq DNA polymerase Master Mix, making a total of 20 μl. PCR reactions in this solution were performed as follows: PCR was utilized to denature DNA templates for 3 min at 40 cycles, including one-minute denaturing at 94 °C, one-minute annealing at 42–50 °C, and angulation at 72 °C for two minutes. To separate the amplified DNA, we first use the Hin 1II(Nlalll) restriction enzyme at 37 °C overnight to digest it and then separate it by electrophoresis on an agarose gel (2%). The genotypes identified from the CAV-1 rs 3,807,992 variant are: uncut homozygous AA (213 bp), cut heterozygous AG (3 bands: 118 and 95 and 213 bp), and cut homozygous GG (2 bands: 118 and 95 bp) [45].

### Physical activity assessment

The International Physical Activity Questionnaire (IPAC) was used to assess physical activity levels. This questionnaire evaluates the number of activities in leisure, housework, work, physical activity related to transportation, and sports in the last seven days. Using the data obtained from this questionnaire, we established metabolic equivalents (METS) and subsequently categorized the level of physical activity of the participants with the following classification: low, below 600 MET/h per week; moderate, 600 to 3000 MET/h per week; and high, more than 3000 MET/h per week [46].

### Assessment of other variables

Economic status, a key covariate in our study, is assessed through a multi-faceted approach. This approach encompasses income levels, employment status, and educational attainment, reflecting a broad spectrum of socio-economic factors. The demographic information was gathered through a structured demographic questionnaire designed to systematically acquire pertinent personal details. Economic status, a focal point of the study, was meticulously assessed through inquiries encompassing annual income and property holdings. Participants were asked to disclose their employment status, educational attainment, marital status, smoking habits, medication history, and any previous experiences with significant trauma. This comprehensive approach ensured a thorough exploration of the participants’ socio-economic and personal backgrounds, contributing to the robustness of the collected data. By incorporating these varied aspects, our study offers a more nuanced analysis of how economic status intersects with dietary choices and metabolic health.

### Statistical analysis

The data were analyzed using IBM SPSS version 23 software. We examined the normality of the data using the Kolmogorov–Smirnov test. Comparison between DII and DIL tertiles and different genotypes of the caveolin gene were evaluated using one-way analysis of variance (ANOVA) and analysis of covariance (ANCOVA). For the relationships, *p* < 0.05 was considered statistically significant. The general linear model (GLM) was employed to investigate the interaction between caveolin gene polymorphisms, DII and DIL. GG was considered as a reference. The genotypes were recorded and given GG, code 0, AG, code 1, and AA code 2. In Model 1. Interaction analysis data were adjusted for age, BMI, and physical activity, and in model 2, economic status, education level, marital status, and job were added. To detect the interaction significance, *P* < 0.1 was considered. The exact test was used for the Hardy–Weinberg Equilibrium (HWE) The Hardy–Weinberg equilibrium and comparison of categorical variables were assessed with the c2 test.

The selection of confounding variables was driven by a thoughtful consideration of factors that could influence the relationships under investigation. Recognizing the complexity of the interactions between dietary indices, genetic variants, and cardiometabolic risk factors, we specifically chose confounding variables that are established contributors to metabolic health [47, 48]. The age and BMI were included as essential covariates given their well-documented associations with both dietary patterns and cardiometabolic outcomes [49]. The total energy intake, a key determinant of nutritional status, was considered to control for variations in overall dietary consumption [50]. Additionally, physical activity was incorporated as a confounding variable, acknowledging its impact on metabolic health and potential to confound the observed associations [51]. This selection aimed to enhance the precision of our findings by accounting for the potential influence of these variables on the relationships of interest.

## Results

### Associations between DII and anthropometric indices, body composition, blood pressure, biochemical factors, and lipid ratios

Participants were divided into three groups based on DII scores: low, medium, and high. Marginally difference was indicated for HDL (*P* = 0.062), and CRI-I (*P* = 0.073). Those in the first tertile had higher HDL and lower CRI-I than those in the third tertile (48.27 ± 9.51 vs. 44.48 ± 10.65) and (3.88 ± 0.85 vs. 4.52 ± 2.24), respectively. After adjusting for age, BMI, total energy expenditure, and physical activity, a significant difference was observed in the job status of the participants (*P* = 0.031). (Table 1).

**Table 1:** Characteristics of the study population across tertiles of DII and tertiles of DIL

Variables	Tertiles of DII			*P*-value	*P*-value	Tertiles of DIL			*P*-value	*P*-value*
	T1 (*n =* 110)	T2 (*n =* 113)	T3 (*n =* 110)			T1 (*n =* 110)	T2 (*n =* 113)	T3(*n =* 110)		
Biochemical parameters										
FBS (mg/dl)	87.42 ± 10.03	87.53 ± 8.75	86.91 ± 10.45	0.91	0.96	87.29 ± 8.81	87.15 ± 11.28	87.40 ± 9.20	0.98	0.74
TC (mg/dl)	182.21 ± 33.76	187.10 ± 35.11	182.47 ± 38.23	0.62	0.48	186.85 ± 34.76	185.46 ± 40.49	179.31 ± 31.52	0.37	0.97
TG (mg/dl)	123.21 ± 62.31	123.07 ± 58.81	115.09 ± 60.36	0.74	0.92	123.31 ± 60.41	122.38 ± 63.97	114.60 ± 56.34	0.75	0.85
HDL(mg/dl)	48.27 ± 9.51^a^	47.41 ± 11.19	44.48 ± 10.65	0.06	0.08	47.53 ± 9.60	46.47 ± 11.56	46.04 ± 10.67	0.65	0.76
LDL(mg/dl)	96.86 ± 23.71	94.95 ± 24.79	91.68 ± 23.11	0.39	0.56	97.47 ± 24.61	94.72 ± 25.30	90.95 ± 21.36	0.22	0.26
Insulin (mIU/ ml)	1.23 ± 0.25	1.27 ± 0.23	1.21 ± 0.22	0.47	0.21	1.23 ± 0.25	1.22 ± 0.22	1.26 ± 0.23	0.70	0.44
Lipid ratios										
CRI-I	3.86 ± 0.82^a^	4.14 ± 1.25	4.41 ± 2.10	0.07	0.04	4.04 ± 0.90	4.29 ± 2.14	4.11 ± 1.29	0.55	0.51
CRI-2	2.06 ± 0.58	2.06 ± 0.55	2.14 ± 0.62	0.62	0.53	2.10 ± 0.56	2.10 ± 0.59	2.06 ± 0.60	0.87	0.90
Blood pressure										
SBP (mmHg)	110.38 ± 11.89	110.36 ± 14.90	112.43 ± 13.31	0.49	0.57	111.64 ± 12.52	110.76 ± 14.71	110.78 ± 12.94	0.88	0.37
DBP (mmHg)	76.62 ± 8.93	77.22 ± 10.10	78.82 ± 9.71	0.28	0.37	78.95 ± 9.30	76.72 ± 9.62	77.01 ± 9.81	0.24	0.01
Demographic variable										
Age (years)	36.46 ± 8.42	36.72 ± 8.81	35.96 ± 8.41	0.82	0.97	36.62 ± 9.01	37.11 ± 7.97	35.41 ± 8.58	0.37	0.17**
Anthropometric variables										
Body mass (kg)	80.57 ± 10.73	79.49 ± 10.64	79.71 ± 11.62	0.77	0.43	78.70 ± 10.61	81.29 ± 11.53	79.77 ± 10.70	0.26	0.19
Height(cm)	161.66 ± 5.32	160.46 ± 5.86	161.85 ± 6.28	0.21	0.28	160.47 ± 5.86	161.42 ± 6.01	162.08 ± 5.59	0.16	0.86
WC (cm)	98.92 ± 9.33	98.31 ± 9.4	98.06 ± 9.52	0.81	0.55	97.44 ± 9.46	99.60 ± 9.17	98.22 ± 9.52	0.28	0.26
BMI (kg/m^2^)	30.77 ± 3.79	30.85 ± 3.68	30.57 ± 3.84	0.87	0.98	30.53 ± 3.86	31.13 ± 3.63	30.54 ± 3.79	0.46	0.27**
WHR (cm)	0.94 ± 0.05	1.90 ± 9.39	0.93 ± 0.05	0.37	0.88	1.92 ± 9.50	0.94 ± 0.05	0.93 ± 0.05	0.36	0.16
FFM (kg)	46.88 ± 5.19	45.98 ± 5.26	46.89 ± 5.74	0.41	0.34	45.75 ± 5.25	46.99 ± 5.73	46.99 ± 5.16	0.19	0.98
SMM (kg)	25.74 ± 3.07	25.21 ± 3.15	25.78 ± 3.40	0.40	0.30	25.12 ± 3.19	25.74 ± 3.34	25.85 ± 3.06	0.24	0.95
BFM (kg)	33.52 ± 7.56	33.34 ± 7.53	33.41 ± 8.05	0.98	0.15	32.70 ± 7.50	34.17 ± 7.56	33.38 ± 8.00	0.42	0.51
Qualitative variable										
Marital status										
Single	15(24.2%)	22(35.5%)	25(40.3%)	0.19	0.24	21(33.9%)	17(27.4%)	24(38.7%)	0.41	0.50
Married	77(35.6%)	72(33.3%)	67(31.0%)			71(32.9%)	77(35.6%)	68(31.5%)		
Physical activity										
Low	38(31.4%)	44(36.4%)	39(32.2%)	0.88	0.62	40(33.1%)	44(36.4%)	37(30.6%)	0.03	0.35**
Moderate	40(35.4%)	38(33.6%)	35(31.0%)			39(34.5%)	37(32.7%)	37(32.7%)		
Intensive	5(45.5%)	3(27.3%)	3(27.3%)			1(9.1%)	9(81.8%)	1(9.1%)		
Education										
Illiterate	1(33.3%)	1(33.3%)	1(33.3%)	0.16	0.10	2(66.7%)	1(33.3%)	0(0.0%)	0.49	0.02
Diploma	19(51.4%)	9(24.3%)	9(24.3%)			15(40.5%)	10(27.0%)	12(32.4%)		
Bachelor and higher	72(30.3%)	84(35.3%)	82(34.5%)			75(31.5%)	83(34.9%)	80(33.6%)		
Economic status										
Poor	5(18.5%)	13(48.1%)	9(33.3%)	0.09	0.52	10(37.0%)	9(33.3%)	8(29.6%)	0.97	0.89
Moderate	50(40.0%)	38(30.4%)	37(29.6%)			42(33.6%)	42(33.6%)	41(32.8%)		
Good	32(28.1%)	38(33.3%)	44(38.6%)			36(31.6%)	38(33.3%)	40(35.1%)		
Job										
Unemployed	59(32.1%)	67(36.4%)	58(31.5%)	0.45	0.03	54(29.3%)	64(34.8%)	66(35.9%)	0.13	0.54
Employed	31(34.4%)	26(28.9%)	33(36.7%)			37(41.1%)	28(31.1%)	25(27.8%)		
Caveolin genotypes										
GG	45(34.1%)	44(33.3%	43(32.6%)	0.98	0.90	46(34.8%)	37(28.0%)	49(37.1%)	0.25	0.35
AG	20(33.3%)	21(35.0%)	19(31.7%)			17(28.3%)	26(43.3%)	17(28.3%)		
AA	24(30.8%)	29(37.2%)	25(32.1%)			28(35.9%)	28(35.9%)	22(28.2%)		

Values are mean ±SD for crude model and mean± SE for adjusted model and qualitative variables are presented as n (%)

Model1 adjusted by age, BMI, total energy intake, physical activity

DII, dietary insulin index; DIL, dietary insulin load; SBP, systolic blood pressure; DB, diastolic blood pressure; BMI, body mass index; FFM, fat free mass; SMM, skeletal muscle mass; BFM, body fat massl; HDL-C, high-density lipoprotein – cholesterol; LDL-C, low density lipoprotein – cholesterol; WC, waist circumference; WHR, waist to hip ratio; FBS, fast blood glucose; TC, total cholesterol; TG, triglyceride; CRI-I, Castelli's risk index-I; CRI-II, Castelli’s risk index-II

*P*-value* obtained from ANCOVA test after adjusted for age, BMI, total energy intake, and physical activity

**_Ihe collinear variable from the GLM (confounder) was not entered_

### Associations between DIL and anthropometric indices, body composition, blood pressure, biochemical factors, physical activity, and lipid ratios:

Participants were divided into three groups based on DIL scores: low, medium, and high. A significant difference was observed in physical activity (*P* = 0.032). After adjusting for age, BMI, total energy expenditure, and physical activity, a significant difference was observed for diastolic blood pressure (DBP) (*P* = 0.014). Individuals in the first tertile had higher DBP than those in the third tertile (79.22 ± 9.2 vs. 77.10 ± 9.21) (Table 1).

### Associations between anthropometric indices, body composition, blood pressure, biochemical factors, and lipid ratios with rs3807992 genotypes

In Table 2, the target population was divided based on different genotypes of the caveolin gene, which include GG (*n =* 88), AG (*n =* 78), and AA (*n =* 167). There was a significant difference between the three genotypes. Participants who carried AA in comparison with other genotypes had a higher height, CRI-I, and FFM *(P* < 0.05) in both the crude and model I (adjusted for age, BMI, total energy intake, and physical activity). In addition, insulin concentrations were higher in GG carriers compared to others (*P* = 0.042). Moreover, after adjusting for potential confounders, a significant link was observed between the genotypes and SMM *(P* = 0.023).

**Table 2:** Characteristics of the study population across rs 3,807,992 genotypes

Variables	Genotypes			*P*-value	*P*-value*
	GG(*n =* 88)	AG(*n =* 78)	AA(*n =* 167)		
Demographic variables					
Age(years)	37.45 ± 9.01	35.58 ± 9.47	36.66 ± 9.25	0.31	0.37**
Anthropometric variables					
Body mass (kg)	79.66 ± 11.66	81.08 ± 12.07	81.85 ± 11.73	0.31	0.38
Height(cm)	160.65 ± 6.14	161.03 ± 5.55	162.30 ± 5.67	0.06	0.04
WC(cm)	98.70 ± 9.70	99.69 ± 10.43	99.49 ± 9.43	0.69	0.84
BMI(kg/m^2^)	31.04 ± 4.28	31.18 ± 3.99	31.02 ± 3.98	0.95	0.94**
WHR(cm)	1.44 ± 6.81	0.94 ± 0.06	0.94 ± 0.04	0.62	0.69
Body composition					
FFM(kg)	46.04 ± 5.72	46.15 ± 5.31	47.60 ± 6.04	0.07	0.01
SMM(kg)	25.24 ± 3.41	25.31 ± 3.15	26.13 ± 3.61	0.08	0.01
BFM(kg)	34.41 ± 8.65	35.16 ± 8.93	34.85 ± 8.92	0.79	0.85
Blood pressure					
SBP(mmHg)	109.40 ± 14.82	111.54 ± 13.26	114.16 ± 15.92	0.09	0.12
DBP(mmHg)	76.88 ± 11.02	77.68 ± 9.24	78.91 ± 10.63	0.42	0.87
Biochemical parameters					
FBS(mg/dl)	87.30 ± 8.95	86.94 ± 7.22	88.48 ± 12.07	0.64	0.45
TC(mg/dl)	186.88 ± 38.07	179.21 ± 32.62	185.80 ± 36.36	0.46	0.64
TG(mg/dl)	121.91 ± 62.26	101.53 ± 49.64	122.98 ± 61.94	0.09	0.11
HDL(mg/dl)	49.51 ± 10.82	44.00 ± 9.82	44.41 ± 10.41	0.00	0.00
LDL(mg/dl)	98.26 ± 26.19	92.31 ± 20.46	92.71 ± 23.14	0.20	0.25
Insulin (mIU/ml)	1.25 ± 0.23	1.16 ± 0.24	1.22 ± 0.24	0.04	0.03
Lipid ratios					
CRI-I	3.92 ± 0.99	4.34 ± 1.33	4.53 ± 2.11	0.02	0.04
CRI-2	2.04 ± 0.56	2.21 ± 0.59	2.20 ± 0.63	0.08	0.09
Qualitative variables					
Marital status					
Single	44 (46.8%)	22 (23.4%)	28 (29.8%)	0.60	0.57
Married	134 (52.1%)	59 (23.0%)	64 (24.9%)		
Physical activity					
Low	57 (48.7%)	25(21.4%)	35(29.9%)	0.30	0.53**
Moderate	58 (52.7%)	19 (17.3%)	33 (30.0%)		
Intensive	2 (20.0%)	4 (40.0%)	4 (40.0%)		
Housing situation					
No home ownership	118 (52.2%)	56 (24.8%)	52 (23.0%)	0.21	0.35
Home ownership	71 (48.0%)	31 (20.9%)	46 (31.1%)		
Family number					
Less than four	160 (51.3%)	71 (22.8%)	81 (26.0%)	0.64	0.65
More than or equal to four	27 (48.2%)	16 (28.6%)	13 (23.2%)		
Smoking					
Yes	16 (59.3%)	5 (18.5%)	6 (22.2%(	0.63	0.26
No	175 (49%)	83 (23.6%)	94 (26.7%)		
Education					
Illiterate	1 (25.0%)	0 (0.0%)	3 (75.0%)	0.15	0.88
Diploma	25 (56.8%)	11 (25.0%)	8 (18.2%)		
Bachelor and higher	152 (50.2%)	70 (23.1%)	81 (26.7%)		
Economic status					
Poor	14 (40.0%)	6 (17.1%)	15 (42.9%)	0.21	0.21
Moderate	82 (52.9%)	36 (23.2%)	37 (23.9%)		
Good	75 (50.3%)	37 (24.8%)	37 (24.8%)		
Job					
Unemployed	120 (50.6%)	52 (21.9%)	65 (27.4%)	0.70	0.75
Employed	68 (49.6%)	35 (25.5%)	34 (24.8%)		

Values are mean ±SD for crude model and mean± SE for adjusted model and qualitative variables are presented as n (%)

The one-way analysis of variance (ANOVA) and the analysis of covariance (ANCOVA) was used for comparison of continuous tertiles of DII and tertiles of DIL, and chi-square for qualitative variables

*P*-value* obtained from ANCOVA test after adjusted for age, BMI, total energy intake, and physical activity

Model1 adjusted by age, BMI, total energy intake, physical activity

*P*-value < 0.05 was considered significant

**The collinear variable from the GLM (confounder) was not entered

^a^LSD test (post hoc), mean the difference between AA and GG

^b^LSD test (post hoc), mean the difference between AG and GG

^c^LSD test (post hoc), mean the difference between AG and AA

### Associations between dietary intake components and DII tertile

As shown in Table 3, except for total fiber, glucose, galactose, fructose, sodium, calcium, and vitamin K, all of the other variables had significant associations among DII groups (*P* < 0.05). After adjusting for total energy, those who had higher DII also had greater intake of carbohydrates, SFA, iron, potassium, magnesium, phosphorus, vitamins E, C, B1, B6, and B9; while the intake of total fat, monounsaturated fatty acid (MUFA), polyunsaturated fatty acid (PUFA), vitamin A, D, B12, and caffeine was lower in compared with other tertiles (Table 3).

**Table 3:** Component of food intakes of study participants across tertile of the DII and tertiles of DIL

Variable	Total (*n =* 333)	Tertile of DII			*P*-value	*P*-value*	Tertile of DIL			*P*-value	*P*-value*
		T1(*n =* 110)	T2(*n =* 113)	T3(*n =* 110)			T1(*n =* 110)	T2(*n =* 113)	T3(*n =* 110)		
Kcal	2605.27 ± 747.03	2557.30 ± 713.59^a^	2436.27 ± 741.14^b^	2823.53 ± 740.09	0.001	–	1863.70 ± 361.36^a^	2573.36 ± 402.19^c^	3371.11 ± 499.79^b^	< 0.001	–
Macronutrients											
Protein(g)	88.20 ± 28.51	86.30 ± 30.20	85.00 ± 28.91	93.30 ± 25.87	0.102	0.293	64.65 ± 16.24^a^	87.79 ± 22.19^c^	111.91 ± 23.99^b^	< 0.001	0.850
Carbohydrates (g)	370.74 ± 119.45	336.12 ± 104.72^a^	347.78 ± 110.38^b^	428.21 ± 121.96	< 0.001	< 0.001	252.31 ± 52.02^a^	363.10 ± 54.43^c^	495.64 ± 87.12^b^	< 0.001	< 0.001
Total fat(g)	94.03 ± 32.49	104.18 ± 35.12^a^	86.56 ± 29.51^c^	91.56 ± 30.37	0.001	< 0.001	71.80 ± 25.93^a^	94.12 ± 25.93^c^	115.95 ± 29.40^b^	< 0.001	< 0.001
Micronutrients											
SFAs(g)	27.98 ± 11.13	28.89 ± 12.33	25.91 ± 9.79	29.16 ± 10.98	0.086	< 0.001	20.10 ± 6.15^a^	27.92 ± 9.22^c^	35.84 ± 11.25^b^	0.340	0.571
MUFA(g)	31.12 ± 11.23	35.31 ± 13.40^a^	28.02 ± 9.09^c^	30.10 ± 9.58	< 0.001	< 0.001	24.93 ± 10.98^a^	31.71 ± 10.57^c^	36.64 ± 8.93^b^	< 0.001	< 0.001
PUFA(g)	19.93 ± 8.51	23.79 ± 10.03^a^	17.73 ± 6.96^c^	18.34 ± 6.94	< 0.001	< 0.001	17.07 ± 9.44^a^	20.08 ± 7.73^c^	22.61 ± 7.41^b^	< 0.001	< 0.001
Minerals											
Sodium(mg)	4238.57 ± 1436.77	4189.57 ± 1434.57	4186.13 ± 1398.55	4340.06 ± 1486.39	0.707	0.096	3281.88 ± 890.94^a^	4234.81 ± 1313.08^c^	5188.78 ± 1371.78^b^	< 0.001	0.920
Potassium(mg)	4309.30 ± 1547.73	4103.90 ± 1453.97^a^	4186.11 ± 1520.02^b^	4637.01 ± 1626.52	0.040	0.180	3099.54 ± 974.99^a^	4314.14 ± 1249.14^c^	5501.16 ± 1349.57^b^	< 0.001	0.753
Calcium(mg)	1160.91 ± 418.63	1103.26 ± 426.48	1147.33 ± 436.08	1231.66 ± 385.80	0.105	0.094	845.14 ± 263.63^a^	1161.80 ± 333.14^c^	1472.38 ± 388.38^b^	< 0.001	0.373
Iron(mg)	18.59 ± 5.90	17.42 ± 5.48^a^	17.52 ± 5.54^b^	20.82 ± 6.09	< 0.001	< 0.001	12.96 ± 2.80^a^	18.54 ± 3.84^c^	24.20 ± 4.36^b^	< 0.001	0.012
Phosphorus(mg)	1627.80 ± 517.59	1532.23 ± 505.03^a^	1576.07 ± 530.04^b^	1774.62 ± 489.46	< 0.001	0.020	1166.48 ± 287.55^a^	1623.51 ± 380.39^c^	2088.49 ± 392.54^b^	< 0.001	0.030
Mg(mg)	455.94 ± 146.76	425.31 ± 139.75^a^	433.78 ± 138.35^b^	508.63 ± 148.90	< 0.001	< 0.001	320.71 ± 84.74^a^	462.71 ± 111.75^c^	582.86 ± 103.63^b^	< 0.001	< 0.001
Zinc(mg)	12.86 ± 4.19	12.24 ± 4.13^a^	12.05 ± 3.88^b^	14.28 ± 4.23	< 0.001	0.062	9.03 ± 2.28^a^	12.87 ± 3.10^c^	16.63 ± 3.03^b^	< 0.001	0.041
Copper(mg)	1.99 ± 0.71	1.87 ± 0.64^a^	1.89 ± 0.61^b^	2.20 ± 0.82	0.002	0.113	1.41 ± 0.36^a^	1.98 ± 0.50^c^	2.57 ± 0.69^b^	< 0.001	0.776
Vitamins											
A(RAE)	771.08 ± 410.09	790.80 ± 425.11	796.56 ± 467.24	725.81 ± 325.00	0.427	< 0.001	594.69 ± 289.24^a^	804.39 ± 449.86^c^	911.90 ± 410.58^b^	< 0.001	0.073
D(mcg)	1.96 ± 1.63	2.16 ± 1.80	2.03 ± 1.70	1.69 ± 1.34	0.129	0.010	1.48 ± 1.04^a^	2.04 ± 1.53^c^	2.36 ± 2.06^b^	< 0.001	0.494
E(mg)	20.86 ± 11.71	15.85 ± 7.85^a^	15.06 ± 6.02^c^	17.24 ± 9.17	< 0.001	< 0.001	15.16 ± 9.57	18.53 ± 10.04^c^	17.99 ± 7.40^b^	0.032	< 0.001
K(mg)	211.30 ± 193.71	205.93 ± 171.61	234.33 ± 254.82	193.33 ± 133.11	0.334	0.122	173.06 ± 112.72^c^	249.73 ± 287.86	210.29 ± 118.66	0.033	0.011
B1 (mg)	2.07 ± 0.65	1.88 ± 0.60^a^	1.95 ± 0.57^b^	2.39 ± 0.66	< 0.001	< 0.001	1.44 ± 0.31^a^	2.05 ± 0.35^c^	2.72 ± 0.48^b^	< 0.001	< 0.001
B2 (mg)	2.19 ± 0.82	2.07 ± 0.78^a^	2.13 ± 0.77^b^	2.38 ± 0.87	0.020	0.151	1.56 ± 0.40^a^	2.17 ± 0.57^c^	2.85 ± 0.83^b^	< 0.001	0.210
B3 (mg)	25.12 ± 9.12	24.11 ± 10.11^a^	23.84 ± 7.94^b^	27.40 ± 8.87	0.012	0.552	18.05 ± 4.74^a^	24.82 ± 6.60^c^	32.41 ± 9.03^b^	< 0.001	0.730
B6 (mg)	2.15 ± 0.71	2.00 ± 0.71^a^	2.13 ± 0.70^b^	2.32 ± 0.68	0.012	< 0.001	1.55 ± 0.38^a^	2.16 ± 0.55^c^	2.75 ± 0.58^b^	< 0.001	0.131
B9 (mcg)	603.44 ± 174.40	573.09 ± 160.75^a^	574.69 ± 170.87^b^	662.53 ± 177.58	< 0.001	0.033	452.22 ± 111.42^a^	601.76 ± 125.06^c^	754.74 ± 133.42^b^	< 0.001	0.073
B12 (mcg)	4.35 ± 2.43	4.36 ± 2.74	4.38 ± 2.70	4.31 ± 1.73	0.980	0.062	3.25 ± 1.43^a^	4.24 ± 1.98^c^	5.56 ± 3.02^b^	< 0.001	0.782
C(mg)	194.30 ± 124.61	161.25 ± 88.81^a^	196.36 ± 108.35^c^	224.91 ± 158.78	0.002	0.010	129.15 ± 79.70^a^	190.88 ± 94.26^c^	262.21 ± 150.80^b^	< 0.001	0.371
Others											
Total fiber(g)	45.15 ± 18.88	45.52 ± 19.67	42.12 ± 18.21	47.85 ± 18.49	0.113	0.614	32.48 ± 13.68^a^	46.07 ± 15.77^c^	56.75 ± 18.52^b^	< 0.001	0.560
Glucose(g)	20.49 ± 11.32	18.52 ± 13.05	20.61 ± 10.19	22.31 ± 10.34	0.074	0.070	13.57 ± 6.84^a^	21.07 ± 12.57^c^	26.74 ± 9.68^b^	< 0.001	0.550
Galactose(g)	2.73 ± 1.91	2.42 ± 1.72	2.72 ± 1.96	3.06 ± 2.00	0.075	0.203	1.98 ± 1.37^a^	2.60 ± 1.69^c^	3.61 ± 2.21^b^	< 0.001	0.333
Fructose(g)	24.85 ± 13.27	22.66 ± 14.75	24.81 ± 12.15	27.04 ± 12.57	0.080	0.201	17.04 ± 9.35^a^	25.36 ± 14.04^c^	32.05 ± 11.51^b^	< 0.001	0.672
Sucrose(g)	32.10 ± 19.81	29.87 ± 17.11^a^	29.30 ± 17.28^b^	37.14 ± 23.57	0.010	0.330	21.12 ± 12.27^a^	32.29 ± 17.59^c^	42.77 ± 22.10^b^	< 0.001	0.791
Caffeine(mg)	150.70 ± 152.18	137.16 ± 99.78^a^	123.31 ± 89.11^b^	192.23 ± 222.95	0.005	0.020	115.52 ± 90.66^a^	153.27 ± 217.93	182.94 ± 108.62	0.012	0.340

Macronutrient and micronutrients adjusted with energy

SFAs, saturated fatty acids; MUFAs, monounsaturated fatty acids; PUFAs, polyunsaturated fatty acids; RAE, retinol activity equivalents

Data are presented as mean ± standard error. The covariance (ANCOVA) was used for comparison of continuous variables among tertiles of DII and tertiles of DIL

^*^Significant level after adjustment for total energy intake. p < 0.05 was considered significant

^a^LSD, mean the difference between tertiles 1 and 3

^b^LSD, mean the difference between tertiles 2 and 3

^c^LSD, mean the difference between tertiles 1 and 2

### Associations between dietary intake components and DIL tertile

After adjusting for total energy intake, a significant association was observed between DIL and intake of carbohydrates, total fat, MUFA, PUFA, vitamin B1, K, E, zinc, magnesium, phosphorus, and iron (*P* < 0.05). This was positively associated with the intake of these components, wherein the individuals with a higher index of DIL had a higher intake (Table 3).

### Interactions between DII and caveolin rs3807992 genotypes on Metabolic components

**Fig. 1:**
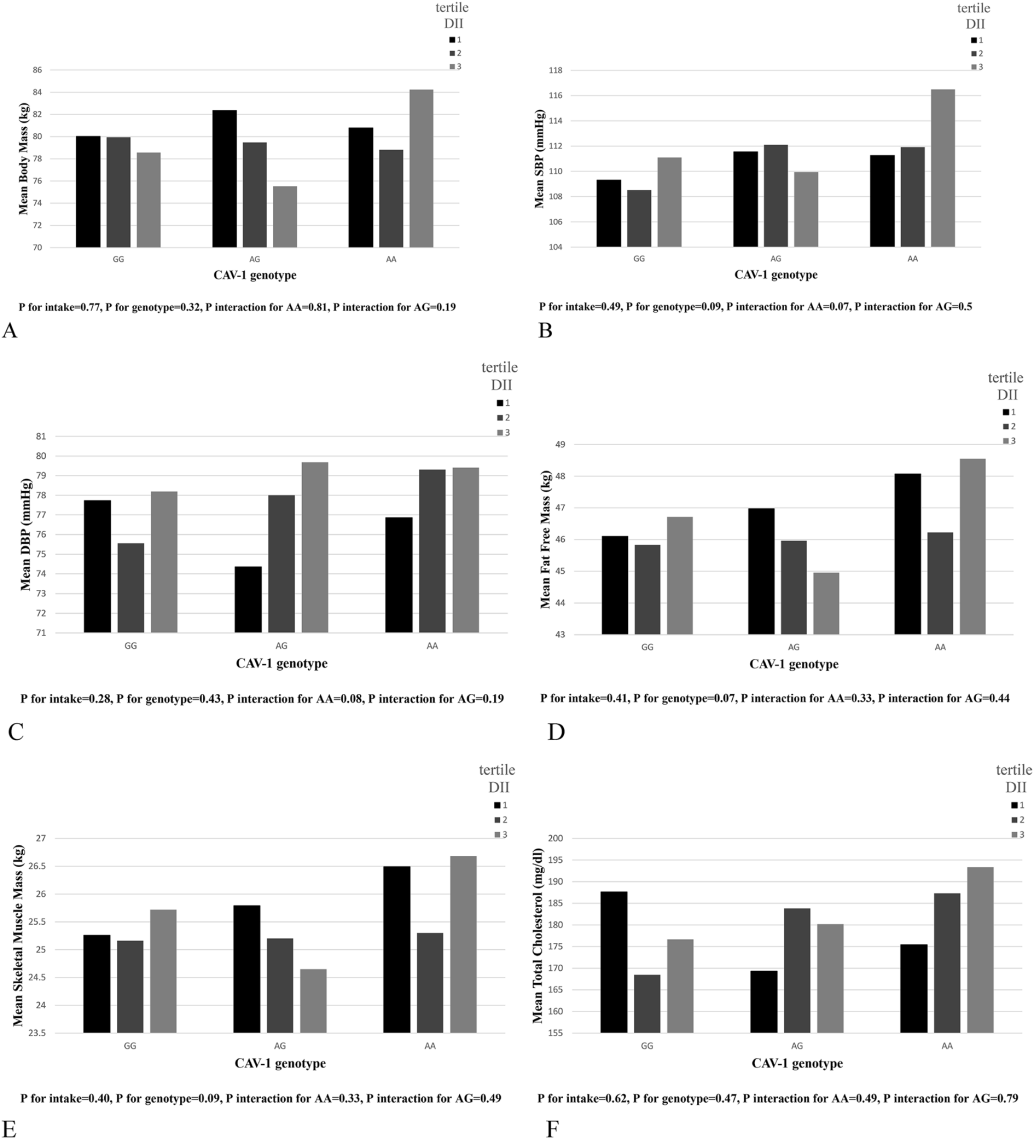

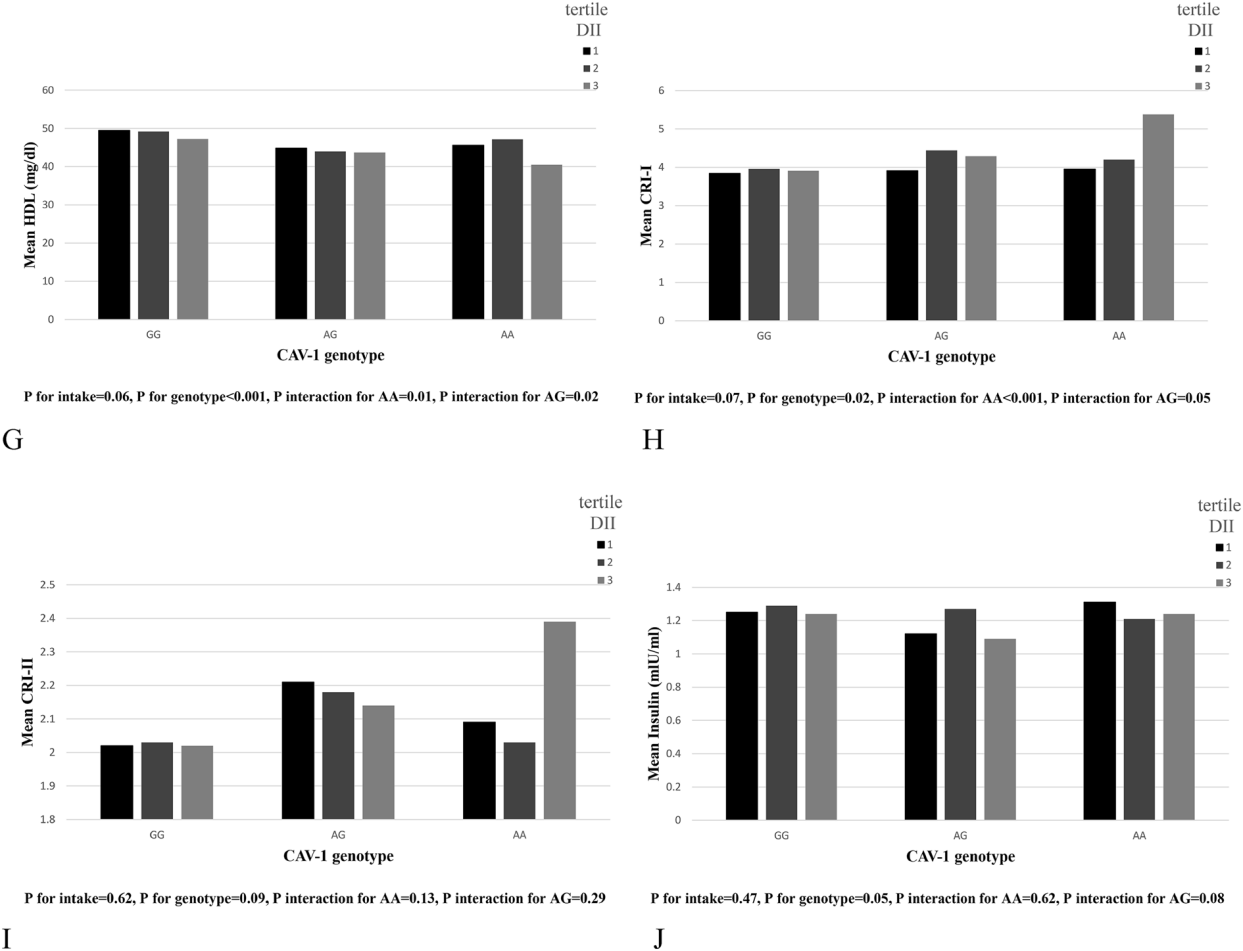
Interaction between Cav-1 genotype with dietary insulin index (DII) on: **A** Body Mass, **B** Systolic Blood Pressure(SBP), **C** Diastolic Blood Pressure(DBP), **D** Fat Free Mass(FFM), **E** Skeletal Muscle Mass(SMM), **F** Total Cholesterol (TC), **G** High-Density Lipoprotein (HDL), **H** CRI-I, **I** CRI-II, **J** insulin

An Allele carrier showed significant interactions with HDL and CRI-I. Participants who scored higher for DII had lower HDL concentrations than the reference population (GG) (genotype AA (*β* = − 2.95, 95% CI (− 5.02, − 0.87), *P* = 0.006) and genotype AG (*β* = − 2.70, 95% CI (− 4.96, − 0.44), *P* = 0.019)). CRI-I indicated a significant interaction with DII in AA and AG genotypes, where the AA genotype (*β* = 0.67, 95% CI (0.34, 1.0), *P* ≤ 0.001), as well as the AG genotype (*β* = 0.41, 95% CI (0.05, 0.77), *P* = 0.024), had a high CRI-I index. In participants with AA genotype and those with higher DII score, significant interactions were observed in body mass (*β* = 1.86, 95% CI (− 0.24, 3.97), *P* = 0.078), systolic blood pressure (SBP) (β = 2.9, 95% CI (0.29, 5.52), *P* = 0.030), diastolic blood pressure (DBP) (*β* = 2.0, 95% CI (0.19, 3.82), P = 0.031), CRI-II (*β* = 0.14, 95% CI (0.01, 0.26), *P* = 0.030), FFM (*β* = 1.10, 95% CI (0.04, 2.17), *P* = 0.042), SMM (*β* = 0.66, 95% CI (0.03, 1.30), *P* = 0.040) and total cholesterol (β = 6.95, 95% CI (− 0.47, 4.37), *P* = 0.067). There was also a significant interaction for insulin in the AG genotype, *β* = − 0.07, 95% CI (− 0.14, − 0.03), and *P* = 0.042 (Table 4 and Fig. 1).

**Table 4:** The interaction of rs 3,807,992 genotypes and DII and DIL on metabolic components

Variables	Model	Genotype	DII			DIL		
			$\beta$	(95%) CI	*P*-value*	$\beta$	(95%) CI	*P*-value*
Body mass (kg)	Crude	AA	0.23	− 1.63, 2.10	0.805	3.03	0.08, 5.97	0.044
		AG	− 1.33	− 3.31, 0.65	0.186	1.05	− 2.10, 4.19	0.514
	Model 1	AA	0.79	− 1.19, 2.77	0.434	4.14	1.04, 7.25	0.009
		AG	− 0.75	− 2.94, 1.43	0.500	2.28	− 1.11, 5.66	0.189
	Model 2	AA	1.86	− 0.24, 3.97	0.078	2.37	0.19, 4.55	0.033
		AG	0.39	− 1.90, 2.68	0.739	1.04	− 1.30, 3.38	0.384
SBP (mmHg)	Crude	AA	2.13	− 0.21, 4.47	0.074	1.55	− 2.12, 5.23	0.408
		AG	0.84	− 1.58, 3.26	0.498	− 0.33	− 4.22, 3.55	0.866
	Model 1	AA	2.43	− 0.05, 4.91	0.055	2.62	1.28, 6.51	0.188
		AG	0.25	− 2.44, 2.93	0.858	− 0.40	− 4.62, 3.82	0.852
	Model 2	AA	2.90	0.29, 5.52	0.030	0.91	− 1.86, 3.68	0.519
		AG	1.04	− 1.77, 3.85	0.460	− 0.82	− 3.72, 2.08	0.579
DBP (mmHg)	Crude	AA	1.51	− 0.18, 3.19	0.079	0.61	− 2.04, 3.26	0.652
		AG	1.17	− 0.57, 2.91	0.187	0.09	− 2.71, 2.88	0.952
	Model 1	AA	1.31	− 0.46, 3.08	0.148	0.82	− 1.97, 3.61	0.564
		AG	0.67	− 1.24, 2.59	0.490	− 0.12	− 3.14, 2.91	0.940
	Model 2	AA	2.00	0.19, 3.82	0.031	− 0.70	− 2.63, 1.23	0.478
		AG	1.53	− 0.42, 3.48	0.125	− 1.03	− 3.05, 0.99	0.318
BMI (kg/m^2^)	Crude	AA	− 0.09	− 0.73, 0.56	0.786	0.41	− 0.60, 1.43	0.425
		AG	− 0.37	− 1.05, 0.31	0.286	0.01	− 1.07, 1.10	0.981
	Model 1	AA	0.06	− 0.61, 0.73	0.858	0.86	− 0.19, 1.91	0.110
		AG	− 0.23	− 0.96, 0.51	0.544	0.48	− 0.67, 1.63	0.416
	Model 2	AA	0.36	− 0.36, 1.08	0.327	0.44	− 0.31, 1.187	0.254
		AG	0.10	− 0.68, 0.89	0.796	0.26	− 0.54, 1.064	0.523
WHR (cm)	Crude	AA	− 0.14	− 1.09, 0.80	0.764	− 0.84	− 2.33, 0.65	0.268
		AG	− 0.15	− 1.15, 0.85	0.768	− 0.87	− 2.46, 0.72	0.284
	Model 1	AA	0.00	− 0.01, 0.01	0.660	0.01	− 0.01, 0.02	0.209
		AG	0.00	− 0.01, 0.01	0.538	0.00	− 0.01, 0.02	0.618
	Model 2	AA	0.01	0.00, 0.02	0.195	0.01	0.00, 0.02	0.065
		AG	0.00	− 0.01, 0.01	0.916	0.00	− 0.01, 0.02	0.414
WC (cm)	Crude	AA	− 0.14	− 1.09, 0.80	0.761	1.70	− 0.82, 4.23	0.186
		AG	− 0.15	− 1.15, 0.85	0.772	0.37	− 2.33, 3.06	0.790
	Model 1	AA	0.55	− 1.13, 2.23	0.519	2.88	0.24, 5.52	0.031
		AG	− 0.44	− 2.29, 1.41	0.644	1.76	− 1.12, 4.64	0.231
	Model 2	AA	1.51	− 0.29, 3.31	0.099	2.00	0.13, 3.86	0.036
		AG	0.40	− 1.55, 2.36	0.685	1.13	− 0.87, 3.13	0.267
BFM (kg)	Crude	AA	0.00	− 1.31, 1.31	0.996	0.61	− 2.04, 3.26	0.270
		AG	− 0.62	− 2.01, 0.77	0.379	0.09	− 2.71, 2.88	0.730
	Model 1	AA	0.47	− 0.89, 1.83	0.499	2.07	− 0.07, 4.21	0.058
		AG	0.08	− 1.42, 1.58	0.920	1.66	− 0.67, 4.00	0.163
	Model 2	AA	1.06	− 0.41, 2.54	0.157	1.41	− 0.11, 2.94	0.069
		AG	0.67	− 0.93, 2.27	0.411	1.19	− 0.45, 2.82	0.156
FFM (kg)	Crude	AA	0.47	− 0.45, 1.39	0.319	2.28	0.84, 3.71	0.002
		AG	− 0.38	− 1.36, 0.59	0.443	1.22	− 0.32, 2.75	0.110
	Model 1	AA	0.59	− 0.41, 1.60	0.248	2.55	0.98,4.11	0.001
		AG	− 0.43	− 1.54, 0.67	0.443	1.26	− 0.45, 2.97	0.149
	Model 2	AA	1.10	0.04, 2.17	0.042	1.29	0.19, 2.39	0.022
		AG	0.16	− 0.99, 1.32	0.785	0.35	− 0.83, 1.53	0.560
SMM (kg)	Crude	AA	0.27	− 0.27, 0.82	0.328	1.32	0.46, 2.17	0.003
		AG	− 0.21	− 0.79, 0.37	0.488	0.72	− 0.19, 1.63	0.122
	Model 1	AA	0.35	− 0.25, 0.95	0.254	1.48	0.55, 2.41	0.002
		AG	− 0.24	− 0.90, 0.42	0.476	0.74	− 0.28, 1.76	0.156
	Model 2	AA	0.66	0.03, 1.30	0.040	0.76	0.10, 1.41	0.024
		AG	0.12	− 0.57, 0.81	0.731	0.21	− 0.49, 0.92	0.553
FBS (mg/dl)	Crude	AA	0.64	− 1.18, 2.45	0.492	0.82	− 2.08, 3.72	0.581
		AG	− 0.31	− 2.23, 1.60	0.748	− 0.61	− 3.70, 2.49	0.701
	Model 1	AA	0.66	− 1.27, 2.59	0.501	0.72	− 2.36, 3.79	0.649
		AG	− 0.19	− 2.32, 1.93	0.859	− 0.56	− 3.96, 2.85	0.749
	Model 2	AA	0.68	− 1.38, 2.75	0.516	0.50	− 1.62, 2.62	0.643
		AG	0.22	− 2.03, 2.47	0.847	0.16	− 2.13, 2.45	0.890
Cholestrol(mg/dl)	Crude	AA	0.64	− 1.18, 2.45	0.865	− 4.30	− 14.84, 6.23	0.424
		AG	− 0.31	− 2.23, 1.60	0.341	− 6.74	− 17.98, 4.51	0.240
	Model 1	AA	3.63	− 3.42, 10.68	0.313	0.72	− 2.36, 3.79	0.414
		AG	0.86	− 6.91, 8.62	0.829	− 0.56	− 3.96, 2.85	0.392
	Model 2	AA	6.95	− 0.47, 14.37	0.067	− 1.76	− 9.38, 5.87	0.652
		AG	0.22	− 2.03, 2.47	0.396	− 4.31	− 12.55, 3.92	0.305
TG (mg/dl)	Crude	AA	0.03	− 0.05, 0.12	0.416	0.03	− 0.10, 0.16	0.635
		AG	− 0.01	− 0.10, 0.07	0.784	− 0.01	− 0.15, 0.13	0.923
	Model 1	AA	0.06	− 0.03, 0.14	0.203	0.06	− 0.08, 0.20	0.370
		AG	0.03	− 0.07, 0.13	0.540	0.05	− 0.10, 0.20	0.531
	Model 2	AA	0.07	− 0.02, 0.16	0.147	0.10	0.00, 0.20	0.047
		AG	0.03	− 0.07, 0.14	0.512	0.07	− 0.03, 0.18	0.171
HDL (mg/dl)	Crude	AA	− 2.41	− 4.28, − 0.54	0.011	− 1.94	− 4.94, 1.06	0.204
		AG	− 2.31	− 4.27, − 0.34	0.021	− 1.97	− 5.18, 1.23	0.227
	Model 1	AA	− 2.46	− 4.37, − 0.55	0.013	− 1.91	− 4.99, 1.17	0.224
		AG	− 2.67	− 4.77, − 0.56	0.012	− 2.26	− 5.66, 1.15	0.194
	Model 2	AA	− 2.95	− 5.02, − 0.87	0.006	− 2.38	− 4.52, − 0.24	0.029
		AG	− 2.70	− 4.96, − 0.44	0.019	− 2.21	− 4.52, 0.10	0.061
LDL (mg/dl)	Crude	AA	− 2.49	− 6.87, 1.90	0.266	− 1.42	− 8.42, 5.57	0.691
		AG	− 2.70	− 7.32, 1.91	0.251	− 2.06	− 9.52, 5.41	0.589
	Model 1	AA	− 2.67	− 7.31, 1.97	0.260	− 2.18	− 9.58, 5.21	0.563
		AG	− 2.13	− 7.24, 2.99	0.415	− 1.47	− 9.66, 6.71	0.724
	Model 2	AA	− 1.52	− 6.30, 3.25	0.532	− 2.55	− 10.04, 4.94	0.505
		AG	− 1.33	− 6.52, 3.87	0.617	− 2.01	− 10.36, 6.34	0.637
CRI-I	Crude	AA	0.48	0.21, 0.75	0.001	0.14	− 0.31, 0.58	0.553
		AG	0.29	0.00, 0.58	0.051	0.01	− 0.46, 0.49	0.957
	Model 1	AA	0.52	0.22, 0.82	0.001	0.14	− 0.35, 0.63	0.584
		AG	0.36	0.03, 0.70	0.032	0.07	− 0.48, 0.61	0.806
	Model 2	AA	0.67	0.34, 1.00	< 0.001	0.36	0.01, 0.70	0.042
		AG	0.41	0.05, 0.77	0.024	0.13	− 0.24, 0.51	0.482
CRI− II	Crude	AA	0.08	− 0.03, 0.19	0.133	0.10	− 0.07, 0.27	0.248
		AG	0.06	− 0.05, 0.17	0.291	0.07	− 0.11, 0.25	0.442
	Model 1	AA	0.08	− 0.03, 0.20	0.170	0.09	− 0.10, 0.27	0.370
		AG	0.09	− 0.04, 0.22	0.101	0.10	− 0.11, 0.30	0.362
	Model 2	AA	0.14	0.01, 0.26	0.030	0.04	− 0.09, 0.16	0.561
		AG	0.10	− 0.03, 0.24	0.137	− 0.01	− 0.14, 0.13	0.917
Insulin (mIU/ml)	Crude	AA	− 0.01	− 0.07, 0.04	0.616	0.01	− 0.08, 0.11	0.756
		AG	− 0.05	− 0.11, 0.01	0.076	− 0.04	− 0.12, 0.05	0.427
	Model 1	AA	− 0.03	− 0.08, 0.03	0.326	− 0.01	− 0.10, 0.08	0.835
		AG	− 0.06	− 0.12, 0.00	0.051	− 0.06	− 0.15, 0.04	0.241
	Model 2	AA	− 0.04	− 0.10, 0.02	0.229	− 0.03	− 0.10, 0.04	0.459
		AG	− 0.07	− 0.14, − 0.03	0.042	− 0.06	− 0.13, 0.01	0.115

Model 1: adjusted for age, BMI, physical activity, and total energy intake

Model 2: MODEL 1+ adjusted for economic status, education level, marital status, Housing situation, number of family members, thyroid status, smoking, and job

GG genotype has 0 risk allele, AG genotype has one, and AA genotype has two risk allele

GG genotype is considered as a reference group

*Significant level in the crude model and after adjustment by Model 1 and 2

For interactions, p < 0.1 was considered significant

### Interactions between DIL and caveolin rs3807992 genotypes on metabolic components

**Fig. 2:**
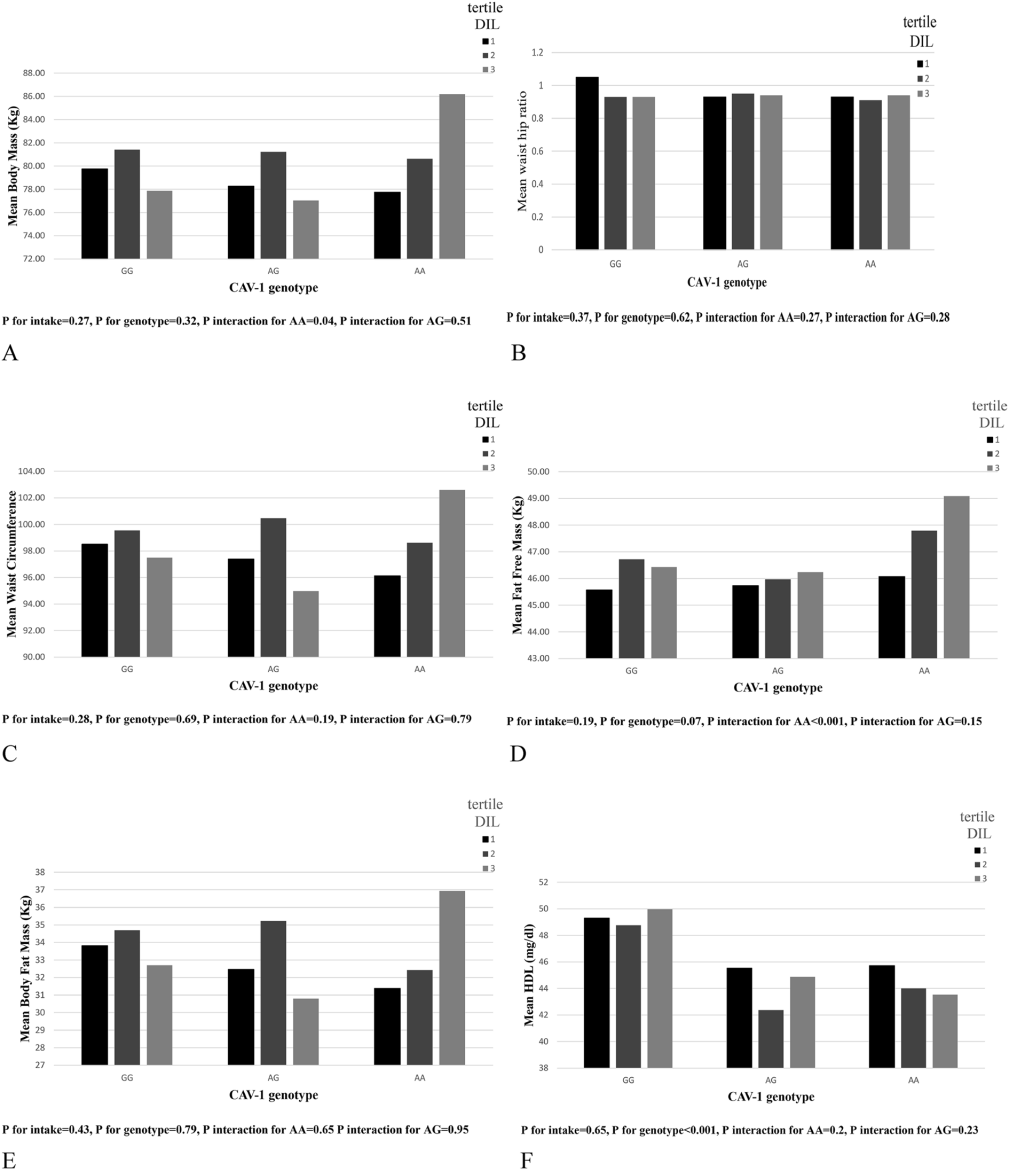

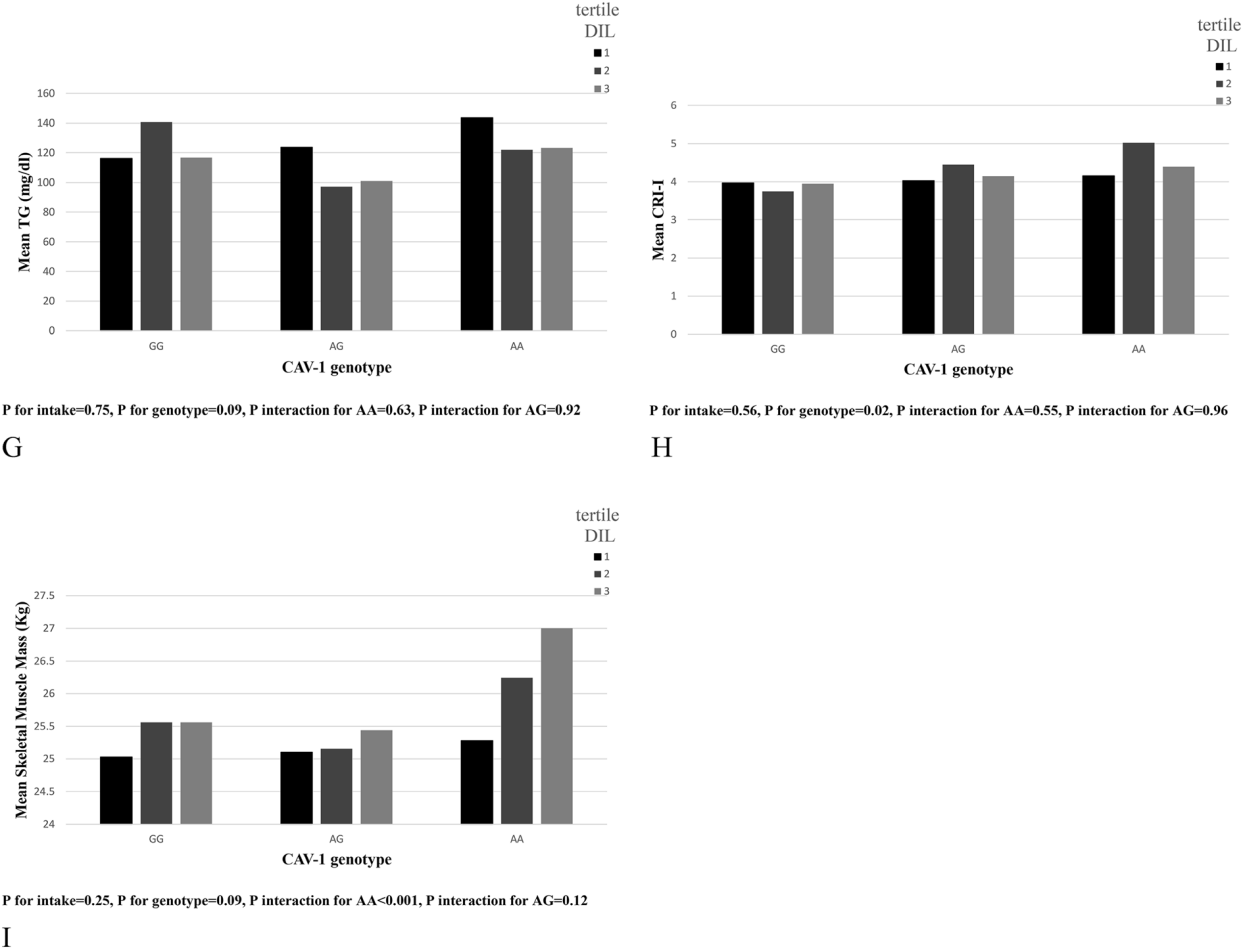
Interaction between CAV-1 genotype with Dietary Insulin Load (DIL) on: **A** Body Mass, **B** Waist Hip Ratio (WHR), **C** Waist Circumference (WC), **D** Fat Free Mass (FFM), **E** Body Fat Mass(BFM), **F** High Density Lipoprotein (HDL), **G** Triglyceride (TG), **H** CRI-I, **I** Skeletal Muscle Mass (SMM)

Those with higher DIL adherence and AA genotype had higher body mass (*β* = 2.37, 95% CI (0.19, 4.55), *P* = 0.033), FFM (*β* = 1.29, 95% CI (0.9, 2.39), *P* = 0.022), and SMM (*β* = 0.76, 95% CI (0.1, 1.41), *P* = 0.024). In addition, we observed interactions for WHR (*β* = 0.01, 95% CI (0.0, 0.02), *P* = 0.065), WC (β = 2.0, 95% CI (0.13, 3.86), P = 0.036), TG (*β* = 0.1, 95% CI (0.0, 0.20), *P* = 0.047) CRI-I (*β* = 0.36, 95% CI (0.01, 0.70), *P* = 0.029)and BFM (*β* = 1.41, 95% CI (− 0.11, 2.94), *P* = 0.069) in individuals with AA genotype, as well as for HDL in those with AG (*β* = − 2.21, 95% CI (− 4.52, 0.10), *P* = 0.061) and AA (*β* = − 2.38, 95% CI (− 4.52, − 0.24), *P* = 0.029) (Table 4 and Fig. 2).

## Discussion

To our knowledge, this is the first study to investigate the interactions of caveolin gene polymorphisms with DII and DIL. Our results indicate that overweight and obese women with elevated DII had lower HDL and higher CRI-I. Moreover, those with higher DIL scores differed significantly in physical activity and DBP. An interaction was observed between DII and caveolin gene polymorphisms on HDL and CRI variables, while another interaction was detected between DII and caveolin on SBP, DBP, FFM, SMM, TC, and Insulin. DIL and rs 3,807,792 CAV-1 polymorphism had a significant interaction on BM, FFM, and SMM variables. In addition, an interaction was observed on WHR, WC, TG, CRI -I, and BFM.

The DII and DIL have recently received substantial attention as these indexes reflect the insulin response associated with different food groups [52, 53]. In this study, participants with higher DII showed lower HDL and higher CRI-I. Nimptsch et al. demonstrated that DII and DIL were not associated with glycemic control and inflammatory markers. At the same time, there were significant positive and negative relationships between TG and HDL with these dietary indexes, respectively. These relationships intensified with increasing body fat [54]. Also, DII and DIL showed a significant negative relationship with HDL among obese people [54]. Given that CRI-I is obtained from the ratio of TC to HDL, we can justify a significant increase in CRI-I as a result of a decrease in HDL.

We also found that individuals who were in the third DIL score tertile had significantly lower DBP than those in the first tertile (77.10 ± 9.21 vs. 79.22 ± 9.2). This may be related to differences in protein intake (Table 3.), as those who had a higher DIL score (third tertile) also reported a higher protein intake (111.91 ± 23.99 g/d vs. 64.65 ± 16.24 g/d). Based on the results of observational studies, high protein consumption is inversely related to blood pressure [55]. Moreover, the previous interventional studies showed that higher protein consumption is associated with a decrease in SBP and DBP [56, 57]. The mechanisms of action are likely related to increases in the intake of arginine (which is a precursor to the vasodilator nitric oxide), biologically active peptides like angiotensin-converting enzyme inhibitors, and foods that are correlated with higher protein consumption as well as lower blood pressure, such as isoflavonoids and soy protein [58].

Based on the previous studies, DII and DIL are associated with anthropometric and metabolic indices. The DONALD cohort (Dortmund Nutritional and Anthropometric Longitudinally Designed Study) measured the relationship between food intake recorded during adolescence and anthropometric indices in adulthood. This investigation found that higher DII and DIL during adolescence are associated with higher body fat percentage in adulthood [59]. However, no such relationship was detected with BMI, hyperinsulinemia, and insulin resistance [59]. In a cohort study with a population of 927 males and females, higher DII was associated with an elevated risk of insulin resistance during a three-year follow-up [15]. High DII stimulates insulin secretion in various ways, such as inhibiting fat oxidation, facilitating carbohydrate oxidation, increasing fat storage, and promoting obesity [60]. Studies have shown that diets that reduce insulin secretion can effectively control chronic diseases and improve body composition [15, 61]. In a study by Sadeghi et al., DII and DIL were associated with a higher risk of metabolic syndrome in women, while no association was observed in men [16]. In agreement with this study, Shoaa et al. concluded that DII was positively associated with abdominal obesity in women [14]. The current study was conducted on women only, and consequently, body composition and appetite may be attributed to gonadal steroids [62]. Indeed, changes in estrogen concentrations may alter hypothalamic pathways, subsequently affecting the production of various hormones that affect appetite [63].

According to our results, the consumption of macronutrients and micronutrients was significantly and positively associated with DII and DIL scores. Participants in our study with greater DII and DIL scores had a higher consumption of carbohydrates, protein, fat, vitamin B1, vitamin B6, magnesium, iron, etc., which was similar to the study of Sadeghi et al. [16]. Carbohydrates are well-established as the main factor in insulin secretion. However, evidence also supports the effects of proteins and fats in this process [17–19]. Indeed, these 3 macronutrients were significantly greater in those who had higher DII and DIL scores. Some minerals and vitamins, such as iron, magnesium, and phosphorus, which were also significant in our results, are also involved in insulin signaling [64]. Vitamin D plays a role in insulin secretion and signaling [65], but participants who had a higher DII score had a lower intake of vitamin D, which is probably due to the limited food sources of this vitamin in Iran [66].

The pathogenesis of the metabolic syndrome and its components may be influenced by interactions between genes and nutrients [67]. In the present study, those with the AA genotype had a significantly greater height, FFM, SMM, and CRI-I and lower plasma insulin concentrations than the population with the reference genotype (GG). Recently, certain genetic strains of CAV-1 have been associated with insulin resistance and hypertriglyceridemia [68]. Increased expression of CAV-1 is linked to the minor allele A [69], and reduced expression of CAV-1 can influence aldosterone and mineralocorticoid receptor signaling in various pathways connected to glycemia and dyslipidemia [70, 71]. Because of the key role of CAV-1 in pancreatic beta cells, it is well established that CAV-1 is involved in energy metabolism disorders such as insulin resistance and hypertriglyceridemia [72].

A significant interaction between HDL and CRI-I variables was observed in the present study between the rs 3,807,992 variant of the caveolin-1 gene and DII. For DIL, the interaction was significant between body composition variables such as body mass, SMM, and FFM. Abaj et al. found that overweight and obese women who carried the A allele had higher BMI, and lower TC, HDL, and LDL. The significant interactions we identified between DIL and the rs3807992 CAV-1 polymorphism about body composition variables, including BM, FFM, SMM, WHR, WC, TG, and BFM, underscore the importance of considering genetic influences when assessing the impact of dietary factors on body composition. Our findings highlight a significant interaction between dietary inflammation, as quantified by DII, and CAV-1 in modulating metabolic health. This interaction sheds light on the complex mechanisms underpinning metabolic disorders in overweight and obese women. The study's implications extend to clinical practice, suggesting that dietary interventions tailored to individual genetic profiles may enhance metabolic outcomes. Furthermore, our research paves the way for future studies exploring the gene-diet interface in metabolic health. In addition, those with higher adherence to a healthy diet pattern had higher HDL and lower hs-CRP concentrations [67], which is consistent with the results of our investigation. In another study by Abaj et al., it was found that consuming more PUFAs could weaken the association between rs 3,807,992 and metabolic syndrome while consuming saturated fatty acids reinforces this association [45]. Diet and nutrients can alter metabolic biomarkers by interacting with caveola-related cellular signaling [73]. Increasing the expression of CAV-1 by reducing the production of nitric oxide (NO) leads to long-term exposure to glucose, which plays an important role in strengthening the inflammatory pathways [74]. Diets with a lower inflammatory load can transport CAV-1 from the caveola to the cytoplasm and impede the inhibitory effects of CAV-1 on endothelial nitric oxide synthase (eNOS) and HDL receptors [75]. Sodium and potassium can also affect the binding of eNOS to the caveola membrane, so changing these two nutrients in diets can also affect the function of CAV-1 [76]. Pojoga et al. found that CAV-1 deficiency was associated with high blood pressure, hyperglycemia, and decreased vasoconstriction [77]. A study showed that CAV-1(+ / +) mice on a high-cholesterol diet had less TC and TG than CAV-1 (- / -) mice [78]. Thus, the expression of CAV-1 can be tightly associated with the intake of some macro and micronutrients in a dietary pattern which may also affect several metabolic components. The observed interactions between DII, CAV-1, and various metabolic parameters, such as HDL, CRI-I, SBP, DBP, FFM, SMM, TC, and insulin, provide insight into the interplay of dietary choices and genetic factors in shaping metabolic outcomes. The interaction between DII and CAV-1 highlights the potential role of dietary insulin response in modulating insulin sensitivity and related metabolic components. Elevated DII scores were associated with lower HDL and higher CRI-I, indicating that the dietary insulin response may play a crucial role in lipid profiles and cardiovascular risk.

CAV-1 is involved in insulin secretion, insulin resistance, and insulin signaling [74] by mediating insulin receptors [79]. These receptors are mainly located in areas of the plasma membrane rich in caveolae and cav-1 and play an important role in insulin signaling and secretion [80, 81]. A homozygous polymorphism in the CAV-1 gene can cause congenital generalized human lipodystrophy type 3 (CGL3), which causes severe IR [82]. CAV-1 variants are also associated with IR [83]. During IR, the insulin receptor detaches from cav-1, and insulin signaling is disrupted, leading to a decreased Glucose transporter type 4 (GLUT 4) transport to the membrane, which reduces insulin sensitivity and glucose uptake [81]. The CAV-1 gene is located in region 7q 31, area 7q 31, and its vicinity is associated with IR, blood pressure, and some vascular conditions [79–86]. The above may be a mechanism for the role of caveolin in hyperinsulinemia. Hyperinsulinemia causes a rise in DII and DIL scores, which logically explains our current outcomes.

In summary, our study contributes to a growing body of evidence highlighting the dynamic relationship between dietary indices, genetic factors, and metabolic markers. Understanding how DII and DIL interact with CAV-1 and impact metabolic outcomes is a crucial step toward more precise dietary interventions and prevention strategies for individuals at risk of metabolic abnormalities. Our findings underscore the potential clinical relevance of this gene-diet interaction and provide a foundation for further research in the field. This was the first study to examine gene interaction with DII and DIL among overweight and obese women. Despite its novelty, our study has certain limitations. Due to the cross-sectional design of the study, any causality cannot be argued. Further studies are needed to determine the exact interaction of the caveolin gene with DII and DIL. Performing clinical trials to establish the effects of diets with low DII and DIL on caveolin gene expression and metabolic components will help expand knowledge on this topic.

## Conclusions

In summary, the results of this study demonstrate that overweight and obese women who had high DII and DIL scores, as well as those who were at risk for the caveolin gene allele (a), had higher body mass, FFM, SMM, TC, and CRI, as well as lower HDL concentrations. In addition, DII had a positive interaction with SBP, DBP, and CRI -II, and a negative interaction with insulin. A positive correlation with WHR, WC, BFM, and TG for DIL was also observed. These outcomes indicate that those who carry the caveolin rs3807992 (A) allele and have greater DII and DIL are at higher risk for cardiovascular disease and metabolic syndrome.

## Data Availability

The datasets analyzed during the current study available from the corresponding author on reasonable request.
